# Evaluation of Cystic Fibrosis Newborn Screening and Follow-Up Process in Georgia (2022–2023)

**DOI:** 10.3390/ijns11020043

**Published:** 2025-06-04

**Authors:** Nino Vardosanidze, Nani Kavlashvili, Lali Margvelashvili, Oleg Kvlividze, Mikheil Diakonidze, Saba Iordanishvili, Dodo Agladze

**Affiliations:** 1 Department of General Pediatrics, Tbilisi State Medical University, Tbilisi 0186, Georgia; 2 General Pediatrics Department, Medical Genetics and Laboratory Diagnostics Center, Tbilisi 0159, Georgia; 3 New Vision University, Tbilisi 0159, Georgia; 4 Department of General Pediatrics, Petre Shotadze Tbilisi Medical Academy, Tbilisi 0144, Georgia; 5 Ilia State University, Tbilisi 0179, Georgia

**Keywords:** cystic fibrosis, genetics, newborn screening, rare diseases

## Abstract

Cystic fibrosis (CF) is a chronic, autosomal-recessive disorder caused by mutations in the CFTR gene, leading to thickened secretions that affect multiple organ systems. This study examines the effectiveness of Georgia’s national CF screening program, which was initiated in 2012 and includes the measurement of immunoreactive trypsinogen (IRT) levels at birth. An analysis of data from 2022 and 2023 revealed a decrease in follow-up attendance for sweat chloride testing among newborns with elevated IRT levels, from 59.9% to 51.2%. The birth prevalence of cystic fibrosis in Georgia varied, suggesting a need to improve both the accessibility of free testing and the quality of follow-up care. Identified barriers include limited access to screening results for pediatricians and the cost of follow-up tests. Recommendations include incorporating free sweat chloride and genetic testing into the national program, as well as improving community education and coordination with social agencies. The identification of 29 CFTR mutations in patients underscores the importance of continued genetic counseling. Overall, while the screening program shows promise, addressing these barriers is essential to improve outcomes and ensure the timely diagnosis and management of cystic fibrosis in Georgia.

## 1. Introduction

Cystic fibrosis is an autosomal-recessive, chronic, progressive, multi-system disorder caused by mutations in the CFTR (cystic fibrosis transmembrane conductance regulator) gene [1,2,3]. This mutation results in the production of defective CFTR protein, which is responsible for sodium and chloride transport across secretory epithelia. As a result, thickened, viscous secretions accumulate in the respiratory system, gastrointestinal tract, reproductive system, etc.

Common symptoms include recurrent respiratory tract infections, malabsorption, endocrine insufficiency, chronic sinus infections, nasal polyps, and infertility [4,5,6]. The disease occurs in 1 in 2500 to 3500 white newborns [7]. The birth prevalence of cystic fibrosis in Georgia was calculated by analyzing data from 2022 and 2023, which showed a birth prevalence of approximately 1 in 5000 in 2022 and 1 in 3769 in 2023, with the identification of 8–10 new cases per year.

Different countries have adopted various newborn screening (NBS) protocols: (1) the IRT-IRT protocol, which involves two rounds of IRT testing followed by additional diagnostic tests if both results are elevated; (2) the IRT/DNA protocol, which combines an initial IRT test with genetic testing to identify CFTR gene mutations; and (3) the IRT/Sweat chloride test—after an elevated IRT result, the infant undergoes a sweat chloride test [8].

The technique used for sweat chloride measurements is the pilocarpine iontophoresis test, which is quantitative. There is only one laboratory in Georgia performing this test, so currently there is no external quality control system in place. Since the program’s initiation, there have been 140 registered patients in Georgia; however, only 84 patients regularly participate in the national monitoring program for rare disorders. Comparable trends have been observed in international CF registries, highlighting gaps in long-term follow-up and program retention [5].

Notably, for unknown reasons, approximately half of the patients with increased immunoreactive trypsinogen levels found through the national newborn screening program did not undergo follow-up tests. In 2022, up to 40.1% of patients did not attend the specialized center for further follow-up and sweat chloride testing, and in 2023, this percentage increased to 48.8%. The national program does not include free sweat chloride testing for specific exclusion, which may contribute to the lack of follow-up among patients. Additionally, limited access to pediatricians for screening results could impact outpatient follow-up for patients with increased IRT levels. The method used was Macroduct^®^ pilocarpine iontophoresis.

Previous studies have outlined the key challenges in cystic fibrosis newborn screening (NBS) programs, including the timeliness of diagnosis and follow-up adherence issues [9,10]. These global observations provide a relevant context for evaluating the current Georgian program.

## 2. Materials and Methods

**Figure 1:**
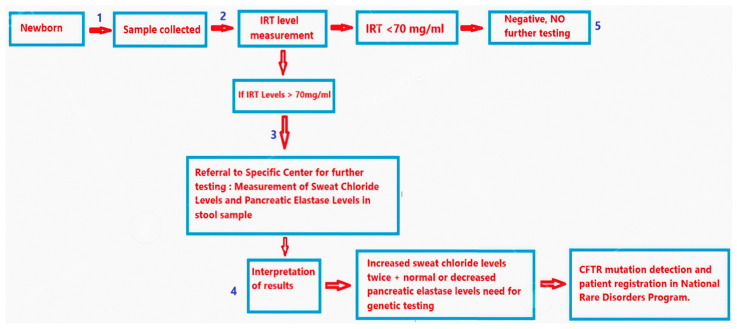
Diagnostic pathway for cystic fibrosis starting from newborn screening.

Georgia’s national cystic fibrosis (CF) newborn screening program was launched in 2012 and is centralized. Blood samples for CF screening are collected within 48–72 h after birth at maternity hospitals. These samples are sent to the “Express Diagnostics” laboratory, where immunoreactive trypsinogen (IRT) levels are measured. If IRT levels exceed 70 ng/mL [8,11], the family is contacted and referred for confirmatory testing, which includes a sweat chloride test and the measurement of pancreatic elastase. The diagnostic pathway is summarized in Figure 1.

Confirmatory sweat chloride tests are conducted exclusively at the “Medical Genetics and Laboratory Diagnostics Center”, the only center in Georgia specializing in rare disorders and host of the national program for the monitoring of rare disorders. It uses the Gibson-Cooke pilocarpine iontophoresis method and is the only test site to do so in Georgia. A sweat chloride level <29 mmol/L is considered normal, 30–59 mmol/L is borderline, and ≥60 mmol/L is diagnostic. A pancreatic elastase level <200 μg/g indicates pancreatic insufficiency. Genetic testing is conducted as a final diagnostic step if clinical suspicion remains.

This study used a retrospective cohort design to analyze data from the national screening program for cystic fibrosis in Georgia for the years 2022 and 2023. In total, data from 790 patients were analyzed as part of this study.

## 3. Results

### 3.1. Birth Data

In total, there were 40,420 live births in 2022 and 37,695 live births in 2023. The data indicate a slight decrease in the number of births from 2022 to 2023 (see Table 1).

### 3.2. New Cases of Cystic Fibrosis

In 2022, eight new cystic fibrosis (CF) patients were identified, with six diagnosed through national screening and two diagnosed late. In 2023, 10 new CF cases were reported, which included 1 missed case and 1 prenatal diagnosis.

### 3.3. Specific Mutations in Georgia

In Georgian cystic fibrosis patients, 29 CFTR mutations were identified. The most common was c.1545_1546delTA (42.7%), followed by W1282X (11.2%). Other mutations included F508del (6.7%). Three novel mutations—c.708dupT, CFTRdele16_17, and c.3170C>G—were reported to the CFTR2 database [12].

## 4. Discussion

In our study, we examined the challenges in and progress of the cystic fibrosis (CF) screening program in Georgia. Georgia has established a well-organized system for CF screening, and as part of this program we routinely measure immunoreactive trypsinogen (IRT) levels in newborns. This protocol, initiated in 2012, aims to facilitate early detection and intervention for newborns at risk for CF. The current approach to cystic fibrosis screening in Georgia follows a multistep process involving IRT screening, sweat chloride testing, and enzyme analysis, with genetic confirmation in unclear cases (see Box 1). This pathway, while structured, still suffers from critical implementation gaps that contribute to missed or delayed diagnoses.

*Theoretically, our national CF NBS program, with IRT determination followed by additional testing (sweat chloride and pancreatic elastase) provides an efficient means for detecting or excluding the diagnosis of CF, but multiple challenges exist. The IRT determination is five times cheaper than the IRT-DNA protocol. Genetic testing can take up to a month with our current capacity in Georgia. But having the screen positive for initial elevated IRT, without further identifying these infants for the additional testing becomes problematic.*

Box 1 **Cystic fibrosis screening and diagnostic pathway in Georgia.**

1. A blood sample should be collected within 48–72 h of birth. Missed cases may occur if one of the following happens:
  (a) The sample was not taken or sent to the lab.
  (b) The sample was not taken within the recommended time frame, especially if the patient was discharged before they were 24 h old.
  (c) The newborn was not fed enterally, leading to false negative IRT levels.
  (d) The blood sample was not properly labeled with the patient’s name and surname.
2. Samples sent to screening lab.
3. A monitoring system is lacking in the government, screening lab, or rare disorders center to ensure patients with elevated IRT levels are referred for sweat chloride testing. As noted in our article, this gap is a key factor in missed diagnoses, as the rare disorders center only received the results after the patient came for additional testing.
4. Normal results: Sweat chloride <29 mmol/L and pancreatic elastase >200 mcg/g. Abnormal results: (a) Sweat chloride >29 mmol/L and elastase >200 mcg/g require the monitoring of chloride levels. If they decrease on retesting, the diagnosis is excluded. (b) Sweat chloride <29 mmol/L and elastase <200 mcg/g call for the initiation of enzyme treatment and monitoring of elastase levels. If there is no response, further tests are conducted to identify the cause of pancreatic insufficiency. (c) Sweat chloride >29 mmol/L and elastase <200 mcg/g indicate high risk for the disease. The sweat chloride test is repeated and genetic testing is performed if levels remain elevated.
5. In the Georgian protocol, if a patient has normal sweat chloride and pancreatic elastase levels within their first month of life, a second sweat chloride test is recommended at 4 months to double-check and exclude the diagnosis of cystic fibrosis.

However, despite the fact that NBS for phenylketonuria (PKU) was initiated in Georgia in 2003, some neonatologists still lack sufficient information about the screening process and its procedures. This gap in knowledge contributes to delays and confusion regarding the screening results. Moreover, it is crucial that every parent at the maternity center is clearly informed that a sample will be taken from their newborn’s heel for genetic testing and that they will need to wait for the results. Unfortunately, many parents are not adequately informed about this process, which creates further uncertainty and misunderstandings.

According to international guidelines, cystic fibrosis cannot be definitively excluded after a single confirmation test; it is considered unlikely only if the diagnostic criteria remain within normal ranges.

Our findings also reveal that attendance for follow-up sweat chloride testing dropped significantly from 2022 to 2023, with only 51.2% of newborns with increased IRT levels attending follow-up appointments in the latter year. This difference is notable and reflects the challenges observed in other countries, where delays in diagnosis and patients lost to follow-up have also been reported [9]. Barriers such as insufficient access to screening results for pediatricians, inadequate parental education, and the absence of free follow-up testing continue to affect the effectiveness of the national program. The lack of a coordinated system to ensure follow-up care for infants with elevated IRT levels suggests a need for greater organizational involvement and clearer governmental responsibility. Since the screening program is government-initiated, we believe that the government’s role in follow-up care should be strengthened. Increased government involvement in overseeing confirmation tests would improve coordination and ensure that more patients attend the necessary follow-up appointments.

One of the critical findings in our study is the low positive predictive value (PPV) of the current CF screening scheme, with only 8 confirmed cases among 460 infants who received follow-up testing (PPV = 1.7%). This low PPV has significant implications for the parental experience, as families are often faced with stressful uncertainty and unnecessary anxiety despite most cases being ultimately considered unlikely to have CF based on the available findings. In addition, the burden of traveling to a single test center and paying out-of-pocket for confirmatory testing may dissuade parents from completing follow-up, especially when the likelihood of a true CF diagnosis appears minimal. Addressing this issue may require refining the screening algorithm or adding DNA testing to increase specificity.

Comparisons to other global NBS models suggest that while Georgia’s IRT-based protocol is cost-effective, it lacks integration with secondary-tier testing such as DNA analysis, which could improve diagnostic accuracy and reduce false positives. As highlighted in recent evaluations of international NBS strategies, economic feasibility and policy adaptability are critical when implementing improvements in resource-limited settings like Georgia [10].

Additionally, a common issue is the false positive results inherent in screening programs. Many parents, based on their experience with previous screenings of other children or in the community, may assume that their child will not have CF, since, in most cases, the diagnosis is excluded rather than confirmed. This leads to a reluctance to pursue additional tests for the exclusion of the diagnosis, as parents may feel that further testing is unnecessary. Addressing this misconception is essential to improving follow-up adherence.

To improve follow-up adherence, we recommend community education on the importance of CF screening and early diagnosis, along with integrating genetic counseling into prenatal and postnatal care. We also propose including free sweat chloride and genetic testing in the national screening program to improve accessibility, follow-up rates, and outcomes. Additionally, ensuring pediatricians have easy access to newborn screening results could support the better management of children’s health.

A key part of our program is identifying CFTR mutations in Georgia. We have found 29 unique mutations, including some new ones, which helps expand our understanding of cystic fibrosis in this population. Comparison with global data from the CFTR2 database helps contextualize the severity and prevalence of these mutations [13].

However, low follow-up rates for genetic testing and counseling show that more efforts are needed to ensure families are informed and involved in managing the condition.

We highlight the importance of better coordinating social services to assist families in navigating the healthcare system. Strengthening these services could improve access to care and increase follow-up compliance.

Despite the progress achieved, our study has limitations, such as potential biases during data collection and the retrospective nature of our analysis. Future research should focus on identifying specific barriers to follow-up care and assessing interventions to improve adherence to testing protocols. In conclusion, while the CF screening program in Georgia has made notable advancements, there is still a pressing need to strengthen follow-up care and community involvement. By addressing barriers to testing, advocating for free sweat chloride and genetic testing in the national program, ensuring pediatricians have easy access to newborn screening results, and improving the support from social agencies, we can greatly enhance the overall effectiveness of CF screening and management in our region.

## Figures and Tables

**Table 1:** Summary of newborn screening and follow-up outcomes for cystic fibrosis, 2022–2023.

	2022	2023
Live Births	40,420	37,695
Increased IRT	768 (1.9%)	645 (1.7%)
Follow-Up Chloride Test	460 (59.9%)	330 (51.2%)
Elevated Chloride	62 (13.5%)	37 (11.2%)
New CF Cases (Late Diagnosis)	8 (2)	10 (1)
Incidence Rate	1 in 5051 newborns	1 in 3774 newborns

## Data Availability

The original contributions presented in this study are included in the article. Further inquiries can be directed at the corresponding author.
